# Stakeholder engagement variability across public, private and public-private partnership projects: A data-driven network-based analysis

**DOI:** 10.1371/journal.pone.0279916

**Published:** 2023-01-06

**Authors:** Shahadat Uddin, Stephen Ong, Petr Matous

**Affiliations:** Faculty of Engineering, School of Project Management, The University of Sydney, Forest Lodge, New South Wales, Australia; University of Greenwich, UNITED KINGDOM

## Abstract

Stakeholder engagement is generally considered one of the most pertinent factors impacting project outcomes. However, there is lacking empirical evidence documenting patterns of stakeholder engagement and their potential differences between public, private and public-private partnership (PPP) projects. This study leverages social network research methods to capture and quantitively compare these engagement structures. Stakeholder network data were collected by an online questionnaire from 17 public, 30 private and 9 PPP projects. A series of network-based analyses were subsequently applied to the data at both the level of individual project stakeholders and entire project stakeholder ecologies. A statistically significant difference (p<0.05) exists among the network-level measures of network size, edge number, density and betweenness centralization across the three project types. Among these four network measures, the density varies significantly (p<0.05) between ‘within budget’ and cost overrun projects for the private and PPP projects. The top-5 stakeholder lists with respect to three node-level centrality values reveal distinctive differences across the three project types. To further interpret the data, exponential random graph models were also applied to determine the most statistically prevalent network motifs within each type of project. Again, statistically significant differences were found across these three project types. The findings consistently point to structural differences in patterns of stakeholder engagement across the public and private domain and illustrate the applicability of network data and analytical techniques to monitoring and managing complex webs of relationships among actors who affect and are affected by diverse types of projects.

## 1. Introduction

Stakeholder engagement is among the core components affecting project performance [1]. Therefore, a better understanding of stakeholders and their engagement drawing from industry precedents will allow project managers to make more informed decisions and follow more appropriate approaches for project success. A stakeholder is an organization or individual involved in a project that is affected by or directly affects, positively or negatively, project execution and, therefore, its outcome [2]. In a project context, stakeholder engagement is the practice that the project authority undertakes to involve different stakeholders constructively in various project activities [3]. A stakeholder network is the structure of relationships among stakeholders. Each node in such a network is a stakeholder. A tie between two nodes indicates that the corresponding stakeholders represented by those nodes have a direct interaction during project execution [2]. Different contexts frequently bring in different patterns of stakeholder interactions. For instance, in some contexts, clients and end-users are the most critical stakeholders within projects, whereas, in others, it may be the engineers who are most central to the project [4]. Stakeholder networks can be considered a footprint of stakeholder engagement at the project level [5]. Such networks provide essential insights into exploring the stakeholder engagement variability across different projects.

Projects can be broadly categorized into three main sectors: private, public or public-private partnership (PPP). The type of sector significantly influences the type of stakeholders involved and the required approach for stakeholder engagement and management. For instance, public projects are often more involved with public diplomacy [6], and an awareness of rules and regulations by diverse stakeholders is of paramount importance within this type of project [6–8]. Stakeholders within such projects require a high level of transparency, and the differences need to be managed meticulously [8]. In contrast, private projects are expected to mainly focus on managing end-user expectations [4, 7]. Limitations of resources have also been highlighted as a relatively more central concern in private sector projects [9]. Although PPP models have recently gained considerable attention for implementing different projects, the overall spending in this sector is still relatively low compared with the public and private sectors [10]. The management of interests in PPP projects is highly complex due to the involvement of diverse public and private stakeholders, with financial and risk implications emerging from this interface [11]. Despite these challenges, PPPs can be lucrative and reduce expenditures if these issues are managed adequately [11].

Stakeholder network structures often vary across public, private or PPP project contexts [1]. These diverging network patterns can be both a cause and a consequence of project leaders’ diverse stakeholder engagement strategies and different external constraints [12]. For instance, public sector actors may be required to follow more publicly transparent and democratic processes. In contrast, private sector actors may engage stakeholders and build supply chain networks under more explicitly profit-driven considerations [7]. Oppong et al. [12] reviewed some critical factors impacting stakeholder networks. They demonstrated an insufficient understanding of the levels of stakeholder engagement variability, especially across private, public and PPP projects. It is here that this study aims to contribute.

In the 1980s, Freeman has proposed that stakeholder theory can help explain how organizations function [13]. Formative research conducted by Donaldson and Preston served to consolidate the burgeoning research on the topic at the time [14]. Studies on the connection and interrelationship between normative ethics and social science at the time also led to research on convergent [15] as well as divergent [16] stakeholder theory. The turn of the century saw stakeholder theory’s increasing application and incorporation in business management. Examples of internal stakeholders in stakeholder theory include employees, customers and suppliers, and external stakeholders include competitors, governments, media and so on. In practice, social movements and social-cause-driven organizations such as Greenpeace have driven further attention to the right of external stakeholders as actors that influence organizations or projects because they are affected by them. Negative relationships with stakeholders were explored [17]. Studies during the 2010s became increasingly interested in the application of stakeholder theory to specific industries. The research has also established that capitalism and ethics are inseparable, and managers often need to account for internal and external stakeholders. Because of this, there exists a level of interplay and complexity between focal organizations and their stakeholders [18–20]. Studies throughout this time became increasingly concerned with sustainable and ethical value creation. Most recent studies have become rooted in sustainability and ethics and advanced the connection between stakeholder theory and strategic management [21, 22].

As summarized above, stakeholder theory has emerged from and was further developed within the fields of general strategic management and organization studies. Projects can be conceptualized as ‘temporary organizations’, which allows extending concepts from more general organization scholarship to the project domain [23]. Via this extension, stakeholder theory has been adapted within project management literature and enjoyed strong attention from a growing community of project researchers [24]. As social network theory became amalgamated with general stakeholder theory as time progressed [2], research of stakeholders in projects has also been connected with formal concepts of social network analysis [25]. This progression allowed to transition from the pre-existing implicit notions of relations between a focal organization or a project and their stakeholders as being independent of each other to fully considering the webs of direct and indirect interdependence between all stakeholders. Following this line of research, numerous studies have demonstrated that network conceptualizations are a viable approach to understanding stakeholder engagement [25–29]. Missonier and Loufrani-Fedida [30] suggested that when approaching stakeholder management in terms of networks, it is crucial to consider the *what*, *how* and *when* factors. It is also to answer this call that analysis of stakeholder networks in relation to their (public or private) context is needed.

In addition to traditional statistical methods, network-based measures and models have become increasingly popular in stakeholder engagement research due to their capability to reveal the core network features. Based on a few network measures, including average path length, density, clustering coefficient and page-rank, Okazaki et al. [31] proposed an approach to capture stakeholder engagement in a stakeholder network. Vance-Borland and Holley [32] analyzed a conservation stakeholder network using a network mapping approach and analysis measures, including density, centrality and centralization. Following a network-based approach, Yang and Zou [33] developed a method for the risk analysis of green building projects. They used network measures of density, cohesion, and centralization to develop this method. Guo and Kapucu [34] used network-level density, compactness, and centralization measures to create and test hypotheses on stakeholder participation in social stability risk assessment. In addition to basic network measures, Pinheiro et al. [35] used exponential random graph models (ERGMs) to explore the role of social capital in resource sharing in collaborative R&D projects. Employment of advanced probabilistic network modelling techniques such as ERGMs has already gained wide acceptance due to their superior ability in identifying dominant network microstructures that play a significant role in shaping up the underlying network to its present state [36].

However, despite this steady stream of work bridging the domains of stakeholder engagement and network research as well as the gained appreciation for the variability in stakeholder engagement across projects reviewed above, there is a lack of systematic empirical evidence of stakeholder network similarities and differences between private, public and PPP engineering projects analyzed with rigorous network research methods. This study aims to contribute to the previous body of network approaches to stakeholder engagement by analyzing a large number of consistently elicited stakeholder networks collected and analyzed with state-of-the-art network analytical techniques. Specifically, we investigate the main stakeholder roles in project networks, overall topological patterns in private, public and PPP contexts, and these metrics’ relation to performance.

## 2. Methods and materials

### 2.1 Data

The data used for this study was of primary nature, collected through an approved survey. Specific criteria were applied for recruiting respondents relevant to the current study. Respondents were targeted among practising project managers to complete an online survey questionnaire on project performance, networks and complexity. The research data collection process had gained ethics approval (Project Number: 2019/794) from the University of Sydney Human Ethics Committee. The ethics committee waived the need for consent from individual respondents since data were collected through an online survey. Anonymity was guaranteed to all participants, and respondents were encouraged to contact and seek clarification regarding any unclear component. The data collected from the respondents have all been de-identified before accessing them for analysis.

An online questionnaire including segments on project performance, project networks and project complexity was designed according to recommended principles from previous work [37, 38] and sent to identified 482 practising project managers. The response rate was 11.62%, providing data from 56 respondents about 56 projects. The aim was not to obtain anything close to a representative national sample but a diverse sample of cases spanning public, private and public-private partnership project strata to illustrate the possibilities of network research methods and enable research for some exploratory comparisons across these different project domains. Respondents were asked about their past medium to large-scale projects and the projects’ stakeholder networks, including an indication of the perceived strength of relationships between stakeholders. In addition to the strength of relations, this study collected role-based information for each project network data (see section 2.4 for details).

A total of 17 public, 30 private and nine public-private-partnership (PPP) project network data were collected, indicating stakeholder engagement structures at the project level. In other sections of the questionnaire, respondents were asked about the project’s cost performance vis-à-vis the planned budget. The design of the survey questionnaire can be found in a S1 File.

### 2.2 Research framework

**Fig 1:**
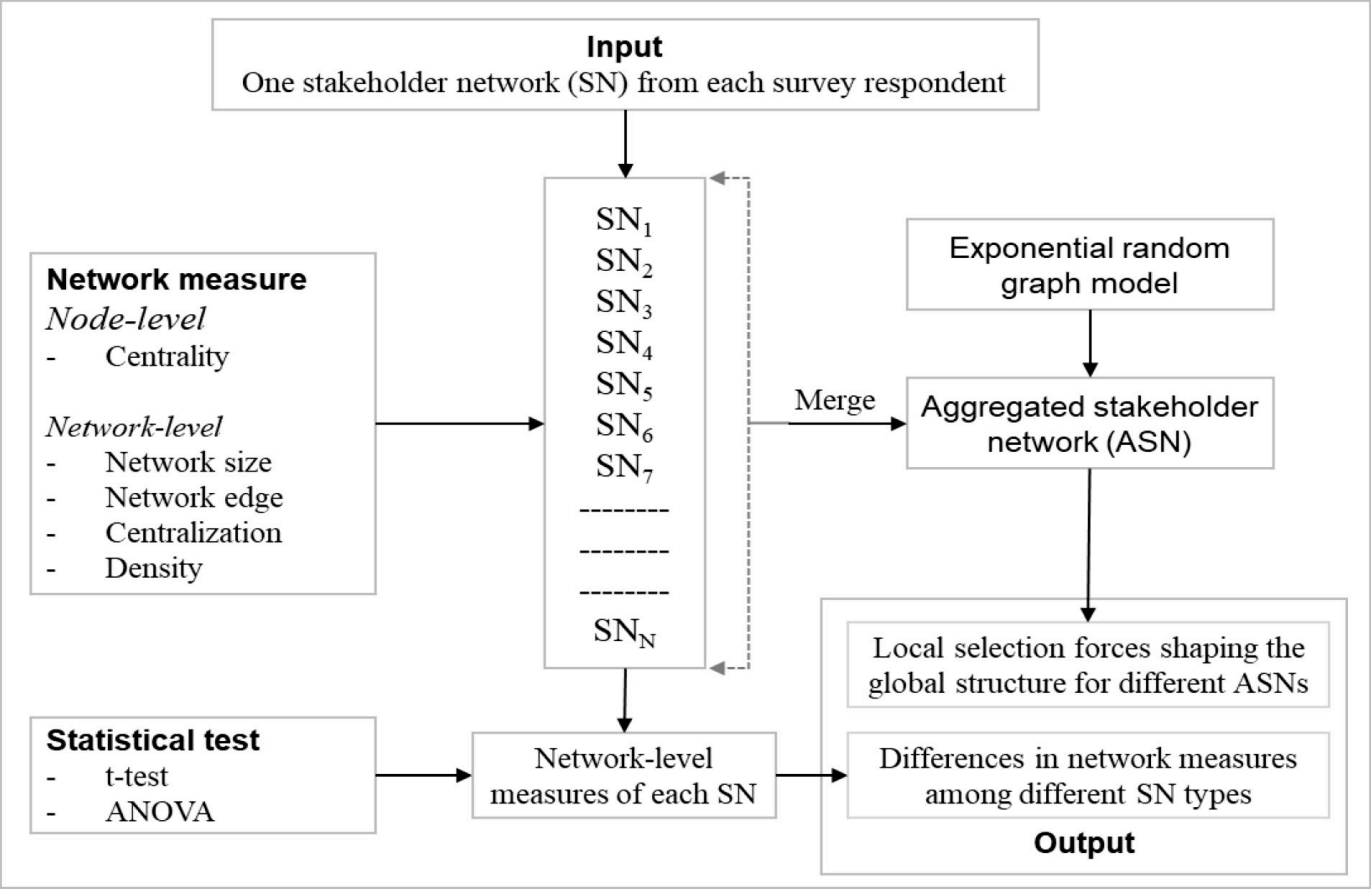
The research framework for investigating the stakeholder engagement variability.

Fig 1 illustrates the research framework used in this study to explore the stakeholder engagement variability across different projects using survey data. The input to this framework is stakeholder networks, one from each survey respondent. Single networks are analyzed using network measures before merging them to create the aggregated network. Exponential random graph models are then applied to explore the aggregated network to find the local selection forces that shape the global network structure. Finally, statistical methods are used for the network-level measures to investigate the differences in their numerical values for different project categories. The following section briefly outlines all network measures and models and the statistical methods used in the framework.

#### 2.2.1 Network measures

This study sets out to understand and interpret data via a select set of network measures and techniques, including node-level measures of centrality, network-level measures of centralization, network density and predominant network motifs identified by the exponential random graph models (ERGM).

Nodes and edges are the fundamental elements for the network representation of any links or connections between the underlying entities [39]. The ideas of node and edge are particularly relevant within this discourse. Specific to the current study, nodes represent the individuals participating in the projects [40]. They have been grouped into role-type as a stakeholder within the project context. Based on the current industry practice and literature [41, 42], this study considered 45 different stakeholder roles for individuals. S1(A) Table details these roles. Edges depict the presence or absence of direct connections between individual nodes. In this study’s context, edges represent the strength of connections between nodes on a scale between 1 and 5. The magnitudes are directly proportional to the connection’s perceived strength by the underlying participants.

For reliability and robustness, three alternative specifications of measures quantifying nodes’ prominence in each project network have been computed (degree, closeness, and betweenness centrality). At the project level, the entire stakeholder network has been examined in terms of its centralization around the most prominent stakeholder roles by analyzing the distribution of the previously computed node-level measures across each aggregated stakeholder network.

**Fig 2:**
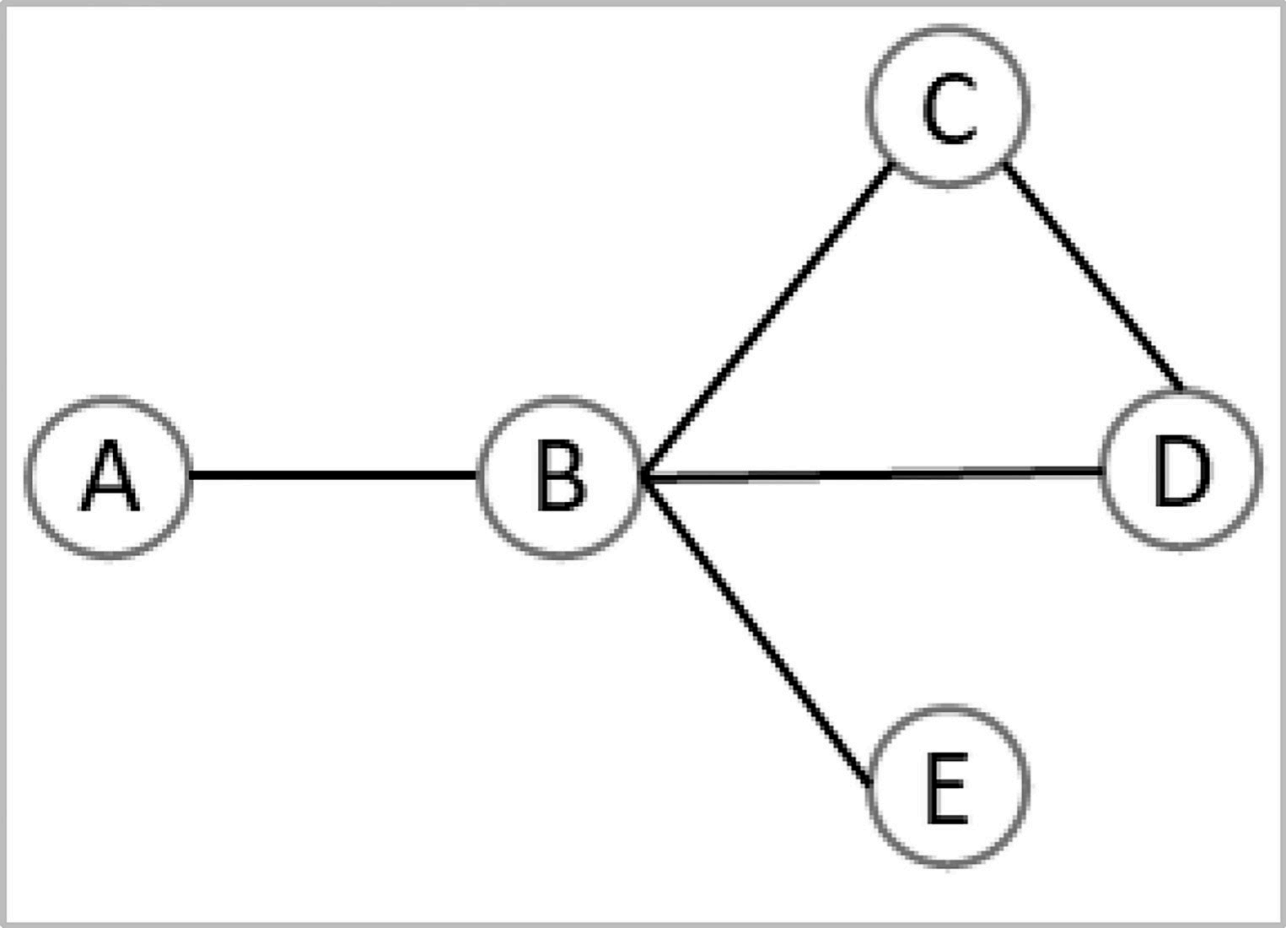
An illustration of a simple network with five nodes and five edges.

*Degree centrality and degree centralization*. Degree centrality is one of the most basic node “importance” measures in a network. It is the number of links connected to a node [40], indicating the networking or communication activity of a stakeholder in a stakeholder network [25]. For instance, node *B* of Fig 2 has a degree centrality of 4 because it directly connects with four other nodes.

The degree centrality of a node can also be normalized to a value between 0 and 1 by dividing it by the total number of other nodes within the network. Here is the relevant equation–

$$C_{D}(n_{i})=\frac{d(n_{i})}{N-1}$$ (1)

Where *C*_*D*_(*n*_*i*_) is the normalized degree centrality of the node *n*_*i*_, *d*(*n*_*i*_) is the number of direct connections it has with the remaining network nodes, and *N* is the total number of nodes. The node *B* of Fig 2 has a normalized degree centrality of 1 $\left(\frac{4}{5-1}\right)$.

**Fig 3:**
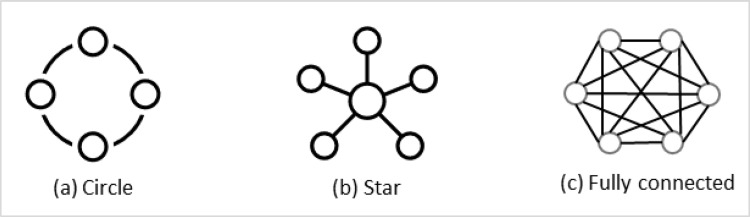
Three prototypical network structures.

While degree centrality is a measure of prominence of individual nodes, degree centralization describes entire networks in terms of the distribution of degrees across the nodes in the network [40]. It indicates how centralized or cohesive nodes are in their degree centrality values. Degree centralization interprets the values in which a minimum value of 0 means that all nodes have an equal level of connection. This is the case of a circular network, as illustrated in Fig 3(A). On the other hand, a maximum value of 1 indicates that one node is in the central position and has a direct connection with the remaining network nodes. These peripheral nodes do not have any connections among them, as in the case of a star network in Fig 3(B). Therefore, the degree centralization value of a dense stakeholder network represents its structural similarity between circle and star networks. High centralization in stakeholder networks suggests that some stakeholder has a disproportionate amount influence compared to others. The following equation is used to measure the degree centralization score (*C*_*D*_) of a network–

$$C_{D}=\frac{\sum_{i=1}^{N}\left[C_{D}(n^{*})-C_{D}(n_{i})\right]}{\left(N-2)(N-1\right)}$$ (2)

Where *C*_*D*_(n*) is the highest degree centrality value among all network nodes, (*N*−2)(*N*−1) is the possible maximum number of edges among the *(N-1)* network nodes (except the central one), and *N* is the total number of network nodes. We first used Eq (1) to calculate the degree centrality of each node of a network and then fed these values to Eq (2) to calculate its degree centralization.

*Closeness centrality and closeness centralization*. Another popular node importance measure is closeness centrality. A node close to other nodes in a network via a small number of network steps has a higher value on this measure [40]. In stakeholder networks, high closeness centrality implies easy access to and the projectability of tacit information and influence to the rest of the project network via a small number of network steps [25]. The following equation quantifies this measure for a node (*n*_*i*_) within a network of size *N*–

$$C_{C}(n_{i})=\frac{N-1}{\sum_{j=1}^{N}d(n_{i},n_{j})}$$ (3)

Where (*n*_*i*_, *n*_*j*_) is the shortest distance between nodes *n*_*i*_ and *n*_*j*_. The node *D* of Fig 2 has a closeness centrality of 0.67 $\left(\frac{5-1}{1+1+2+2}=\frac{4}{6}\right)$.

The network-level extension of closeness centrality is closeness centralization. This value is calculated through comparisons with the maximum observed closeness centrality value [40]. For a network with a size of *N*, the following equation can capture its closeness centralization (*C*_*C*_)–

$$C_{C}=\frac{\sum_{i=1}^{n}\left[C_{C}(n^{*})-C_{C}(n_{i})\right]}{\left[\frac{\left(N-2)(N-1\right)}{2N-3}\right]}$$ (4)

Where *C*_*C*_(*n*_*i*_) represents the closeness centrality of node *i*, quantified by Eq (3), and *C*_*C*_(n*) represents the highest closeness centrality among all nodes. The computed value lies between 0 and 1, inclusive. When all nodes have an equal closeness centrality value, this measure will score 0. This happens when each node has a direct connection with all other remaining nodes, as in the case of a fully connected network illustrated in Fig 3I. This measure has the highest value of 1 for a star graph (Fig 3(B)) as with the degree centralization.

*Betweenness centrality and betweenness centralization*. The last highly popular node centrality measure used in this study is betweenness centrality, which quantifies to what extent a node lies on the shortest pathways between other network nodes, potentially indicating a broker position within a network [40]. Betweenness centrality reflects a stakeholder’s potential to control the overall communication flows in a stakeholder network [25]. Mathematically, it can be represented by the following equation, where *g*_*jk*_ represents the number of shortest paths connecting nodes *j* and *k*. *g*_*jk*_(*n*_*i*_) is the number of shortest paths between *j* and *k*, which include node *i* and *N* is the total number of nodes within the network.


$$C_{B}(n_{i})=\frac{\sum_{j<k}\frac{g_{jk}(n_{i})}{g_{jk}}}{\frac{\left[\left(N-1)(N-2\right)\right]}{2}}$$ (5)

Node *A* of Fig 2 has a betweenness centrality of 0 since it does not position in the shortest path of any other pair of network nodes.

Betweenness centralization is a network-level measure that extends on betweenness centrality. The value is computed relative to the highest observed betweenness centrality value. With reference to Eq 6 for the betweenness centralization (*C*_*B*_) of a network with *N* nodes, *C*_*B*_(n*) represents the largest observed betweenness centrality value, and *C*_*B*_(*n*_*i*_) represents the determined betweenness values, quantified by Eq (5), for each node.


$$C_{B}=\frac{\sum_{i=1}^{n}\left[C_{B}(n^{*})-C_{B}(n_{i})\right]}{\left(N-1\right)}$$ (6)

The value for the betweenness centralization also lies between 0 and 1. For a star network (Fig 3(B)), this measure takes its maximum value of 1. For a circular network (Fig 3(A)), this measure scores 0.

*Network density*. For a given network, the density is the ratio between its number of edges and the maximum possible number of edges that it could have among its nodes [40]. In a fully connected network (FiI(c)), the density is 1. For a network with *N* nodes and *e*_*t*_ edges, the following equation measures its density value–

$$Density=\frac{{2\times e}_{t}}{N(N-1)}$$ (7)

The network density in Fig 2 is 0.50 since it has only five edges out of the ten possible edges among its five nodes. In stakeholder networks, high network density indicates a higher level of communication and interaction among stakeholders and possibly a high potential for coordination and emergence of project-related norms as well as generalized sharing of tacit knowledge.

#### 2.2.2 Statistical methods

*Independent sample t-test*. For comparing project cost performance (i.e. within budget versus cost overrun) in relation to project stakeholder network structure, we used the independent sample t-test. The following equation measures the *t-*value for two sets of scores–

$$t=\frac{{\bar{X}}_{1}-{\bar{X}}_{2}}{\sqrt{\left(\frac{s_{1}^{2}}{N_{1}}+\frac{s_{2}^{2}}{N_{2}}\right)}}$$ (8)

Where ${\bar{X}}_{1}$ and ${\bar{X}}_{2}$ are the mean of two sets of numbers, respectively. Similarly, s and N represent the standard deviation and the size of the number set, respectively. The higher the *t* value is, the more likely it is that there is a statistically significant difference between the mean values of the underlying number sets. This test can compare the mean values of only two groups. We used the IBM SPSS statistics software tool to conduct the t-test [43].

*One-way ANOVA*. Analysis of variance (ANOVA) is a techniquenalysesalyzes the difference among the means of more than two groups. We considered one-way ANOVA to compare the mean values for different network-level measures across public, private and PPP networks. The *F*-score quantifies this measure–

$$F=\frac{{SS}_{between\_group}\times {df}_{within\_group}}{{SS}_{within\_group}\times {df}_{between\_group}}$$ (9)

Where *SS* and *df* stand for the sum of squares and the degree of freedom, respectively. A higher *F-*value indicates that the difference among the mean values across groups is more likely to be statistically significant.

#### 2.2.3 Network merging

**Fig 4:**
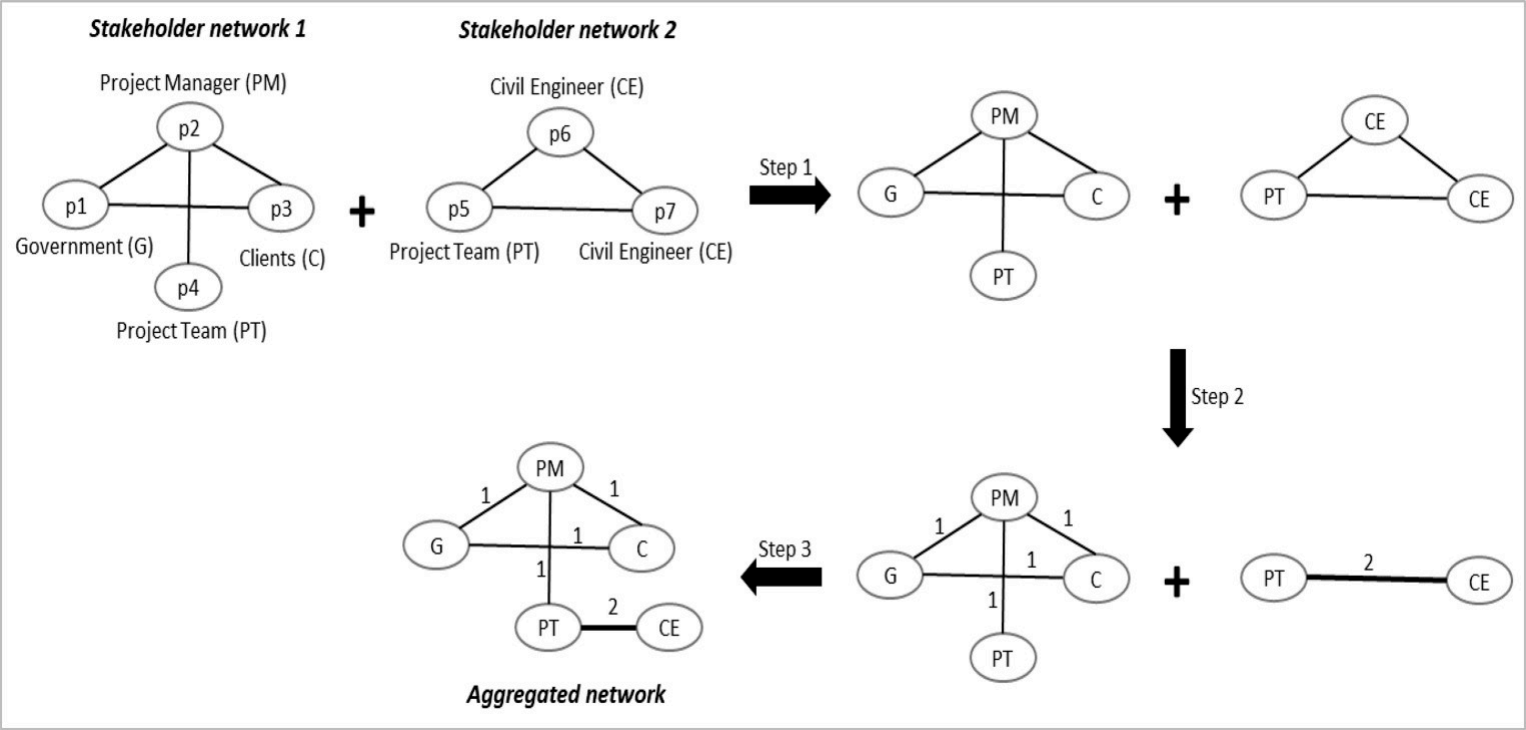
Network merging process. Edge thick in the aggregated network proportionates to the edge weight.

In addition to exploring each of the 56 networks using network measures and statistical methods, we constructed and analyzed three aggregated stakeholder networks for each project category (public, private and PPP). We merged 17 public networks, 30 private networks and nine PPP networks into aggregated public, private, and PPP networks, respectively, one each. Fig 4 illustrates the steps of this merging process based on the data from this’study’s two public networks. The first public network consisted of four people (*p1* to *p4*). They belonged to four different roles. Three people (*p5* to *p7*) were in the second network and belonged to two different roles. The text box next to each node denotes its stakeholder type. We considered 45 different stakeholder types, as outlined in S1(B) Table. Although these seven people are different individuals, there are commonalities between their roles (i.e., stakeholder types).

The first step replaces the node names with their stakeholder roles. Edge weights are determined in the second step. Duplicate stakeholder roles are removed in this step, and edge weights are quantified accordingly. After this step, the second stakeholder network has become a network with two nodes since the civil engineering node appears twice in the previous step. Accordingly, the civil engineer node has an edge weight of 2 with the project team node. Two role-based networks from the second step are merged in the third step to create the aggregated network. The nodes in the aggregated network are the stakeholder type, not individuals, as in its constituent networks. There are five nodes in the aggregated network since the seven individuals of the two constituent networks belong to one of these five stakeholder types. There is an edge with a weight of 2 between the project team and civil engineer in the aggregated network. This edge is from the second network.

#### 2.2.4 Exponential random graph model

We used exponential random graph models (ERGMs) to identify local patterns that shaped the global structure of the three aggregated stakeholder networks resulting from the network merging process. ERGMs are statistical models primarily utilized to study network composition from underlying locally prominent microstructures, such as 2-star and triangles [36, 44]. For an observed network, they can effectively determine the local patterns that play a statistically significant role in reaching its present global structure. In the stakeholder context, these models can explore the micro-level role-based stakeholder communications that shape the observed structure of the underlying aggregated stakeholder network. For example, the dyadic tie between actor pairs could influence reaching the observed structure of an aggregated stakeholder network. If this observed aggregated network is from projects with positive attributes (e.g., on-time completion), then increased interactions between stakeholders can be recommended for better project outcomes. Or, while controlling for density of ties and prevalence of other micro-structures by ERGM, if star structures are found to be statistically significantly under-represented in successful projects, it can be inferred that decentralized stakeholder network structures are associated with positive project outcomes. Based on the network fundamental parameters (e.g., the number of nodes and edges) of an observed network, there can be many possible global network structures (*X*), and the observed one (*x*) is one of them. An ERGM detects the microstructures that play a significant role in simulating the observed network state. The following is the underlying equation of ERGMs–

$$\mathrm{Pr}\left(X=x)=\frac{1}{k}Exp\;\{\sum_{A}\eta_{A}g_{A}(x)\right\}$$ (10)

Where *k* is a normalizing constant to ensure the proper probability distribution. The summation is over different microstructures or configurations. η_*A*_ is the parameter for configuration *A*, and *g*_*A*_(*x*) is the network statistics in the observed network (*x*) for *A*. If *A* represents the configuration of an edge, for example, then *g*_*A*_(*x*) will denote the number of edges in the observed network (*x*).

**Fig 5:**
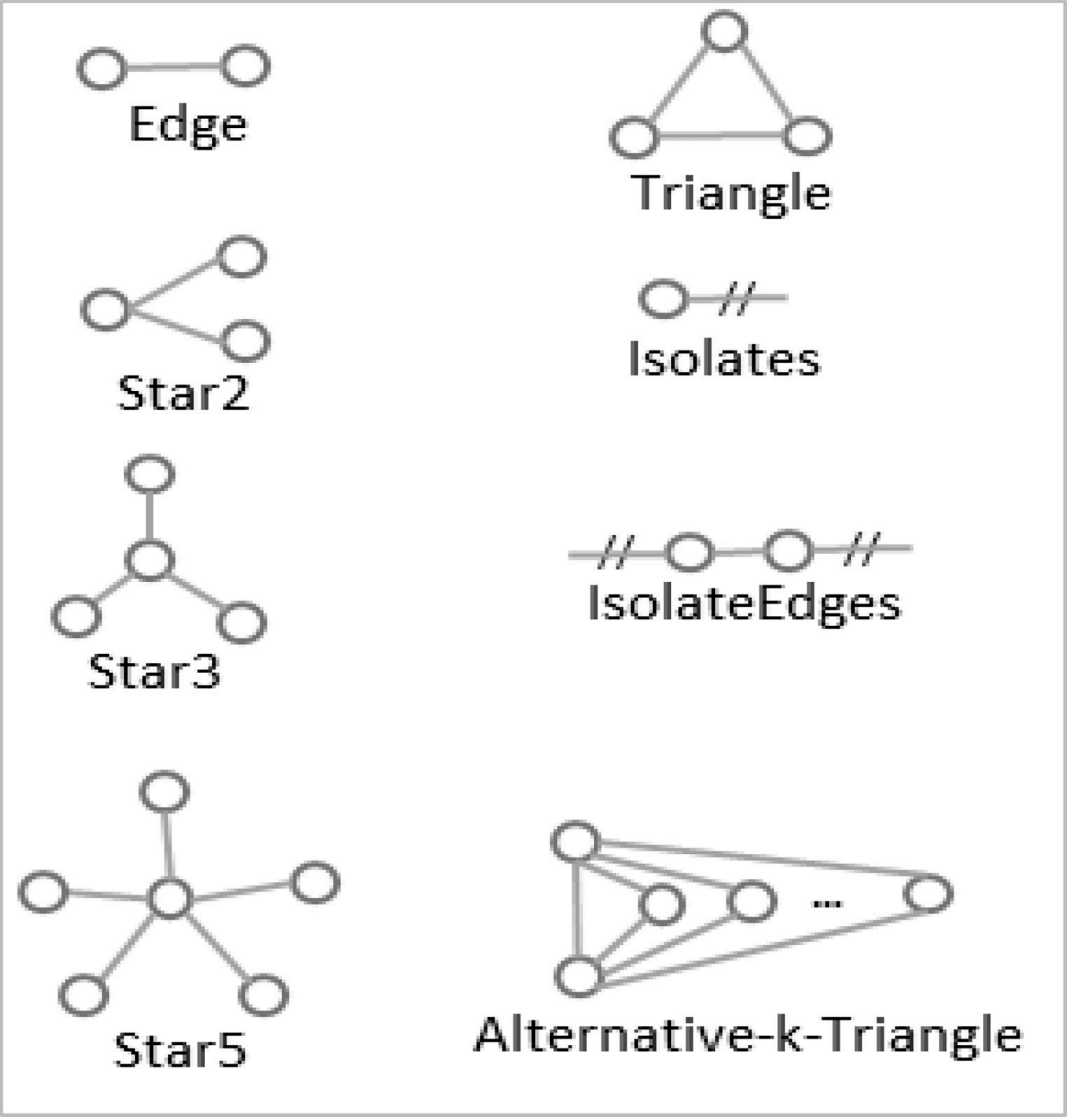
Microstructures or configurations of the exponential random graph model are considered in this study.

There are many possible microstructures or configurations to consider to model on a given network using ERGMs. Out of all possibilities, this study considered the eight structures shown in Fig 5. We used the MPNet software tool [45] to implement ERGMs for our three aggregated networks.

## 3. Results

### 3.1 Basic network statistics

Each survey response provided network data for stakeholder engagement for the corresponding project. Therefore, we have the same number of stakeholder network data from 56 survey responses. Table 1 reports the basic statistics of network-level measures for all stakeholder networks. On average, public networks have the highest number of nodes and edges, followed by PPP networks. Interestingly, the maximum density value for all three types of networks is 1. A network has a density of 1 when all possible node pairs have a direct direction between them, as in the case of a fully connected network. That means at least one network from each of the three categories is fully connected. For such a fully connected network, each node’s three centrality values (degree, closeness and betweenness) will be equal, which will make the corresponding centralization values 0. For this reason, the minimum centralization value is 0 for each of its three variants across all network types, another notable observation within the network-level measures.

**Table 1:** Basic network statistics of the 56 stakeholder networks resulting from the survey response data used for this study.

Items	Public project	Private project	Public-private partnership project
#. stakeholder networks	17	30	9
Network size			
*Minimum*	4	3	5
*Maximum*	15	10	12
*Average*	7.88	5.87	7.44
*Standard deviation*	3.66	1.85	2.96
Edge number			
*Minimum*	5	3	3
*Maximum*	66	45	66
*Average*	24.24	11.87	17.33
*Standard deviation*	20.57	8.02	22.41
Density			
*Minimum*	0.10	0.29	0.16
*Maximum*	1.00	1.00	1.00
*Average*	0.83	0.83	0.58
*Standard deviation*	0.25	0.24	0.35
Degree centralization			
*Minimum*	0.00	0.00	0.00
*Maximum*	0.47	0.76	0.58
*Average*	0.12	0.13	0.24
*Standard deviation*	0.17	0.21	0.22
Closeness centralization			
*Minimum*	0.00	0.00	0.00
*Maximum*	0.54	0.77	0.64
*Average*	0.15	0.17	0.29
*Standard deviation*	0.21	0.25	0.25
Betweenness centralization			
*Minimum*	0.00	0.00	0.00
*Maximum*	0.50	0.82	0.71
*Average*	0.09	0.10	0.30
*Standard deviation*	0.16	0.19	0.27

### 3.2 Top-5 stakeholders

Table 2 shows the top-5 stakeholder roles that appeared most frequent across the surveyed networks. These appearance data of relative frequency are statistically significant at p<0.001 when compared with the values from 45 stakeholder roles considered in this study using a t-test. Two stakeholder roles (construction manager and architects) are in the top-5 list of all three types of projects. Besides these two stakeholder roles, there are two other common types of stakeholder roles (clients and project team) in the top-5 lists of public and private projects and one common stakeholder (project manager) between the top-5 lists of private and PPP projects.

**Table 2:** Top-5 nodes that appeared most in the three different types of stakeholder networks. The proportion of percentage appearance in parentheses follows the count value. All values in this table are significant at p<0.001, resulting from a t-test.

Rank	Public		Private		Public-private partnership	
	*Stakeholder name*	*Appearance (%)*	*Stakeholder name*	*Appearance (%)*	*Stakeholder name*	*Appearance (%)*
1	Clients	10 (59%)	Project Manager	16 (52%)	Architects	4 (44%)
2	Project Team	10 (59%)	Architects	14 (45%)	Environmental Regulators	4 (44%)
3	Construction Manager	8 (47%)	Project Team	13 (42%)	Government	4 (44%)
4	Project Sponsor	8 (47%)	Clients	11 (35%)	Project Manager	4 (44%)
5	Architects	6 (35%)	Construction Manager	11 (35%)	Construction Manager	3 (33%)

### 3.3 One-way ANOVA results

We conducted a one-way ANOVA test to further investigate whether the basic statistics reported in Table 1 are statistically different across three different project types. The test results are reported in Table 3. Four measures (network size, edge number, density and betweenness centralization), out of six, show statistically significantly different values at p<0.05 across the three different project types. The remaining two measures (degree centralization and closeness centralization) do not display any statistically significant difference.

**Table 3:** Results from the one-way analysis of variance (ANOVA) test for the various network measures across three different project categories.

		Sum of squares	df	Mean square	F	Sig.
Network size	Between groups	49.53	2	24.76	3.42	0.040
	Within groups	383.45	53	7.24		
Edge number	Between groups	1667.49	2	833.74	3.49	0.038
	Within groups	12652.54	53	238.73		
Density	Between groups	0.48	2	0.24	3.47	0.038
	Within groups	3.66	53	0.07		
Degree centralization	Between groups	0.09	2	0.05	1.11	0.336
	Within groups	2.13	53	0.04		
Closeness centralization	Between groups	0.13	2	0.06	1.12	0.335
	Within groups	2.99	53	0.06		
Betweenness centralization	Between groups	0.32	2	0.16	4.19	0.020
	Within groups	2.02	53	0.04		

**Fig 6:**
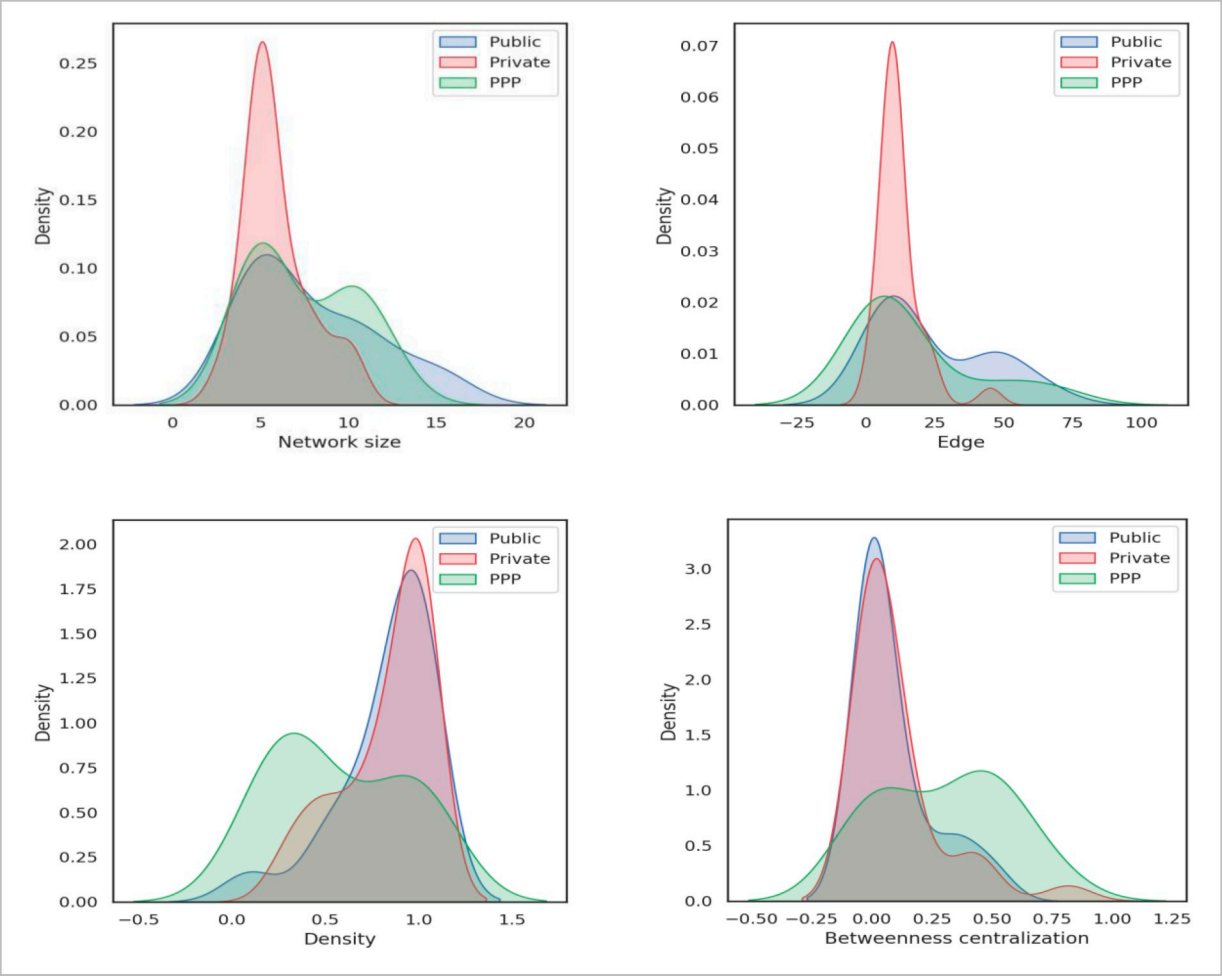
The kernel density estimations of four network measures.

Fig 6 illustrates the kernel density estimations (KDEs) of these four measures. In statistics, KDE is used to estimate a variable’s probability density function [46]. There are noticeable differences across these KDEs of this figure, echoing the findings reported in Table 3.

### 3.4 Independent sample t-test results

We then investigated the impact of the four network-level measures (network size, edge number, density and betweenness centralization) that revealed statistically significantly different values at p<0.05 across the three different project types (see Table 3) on project cost performance. We used an independent sample t-test for this purpose, and the results are reported in Table 4. We categorized each project into two groups based on whether it was finished within the allocated budget. As can be seen in Table 4(A), there is no significant difference in these four measures between the two types of public projects. On the other side, as revealed in Table 4(B) and 4(C), the density measure shows a statistically significant difference at p<0.05 for both private and PPP project categories.

**Table 4:** Results from the independent sample t-test for four network measures. Each project is grouped based on its cost performance (within budget or not).

(a) Public projects						
	Within budget	N	Mean	Std. Deviation	t	Sig.
Network size	Yes	6	7.67	3.39	-0.183	0.858
	No	11	8.00	3.95		
Edge number	Yes	6	30.00	25.35	0.762	0.468
	No	11	21.09	18.03		
Density	Yes	6	0.87	0.20	0.524	0.608
	No	11	0.81	0.29		
Betweenness centralization	Yes	6	0.07	0.15	-0.422	0.681
	No	11	0.10	0.17		
(b) Private projects						
	Within budget	N	Mean	Std. Deviation	t	Sig.
Network size	Yes	11	5.27	1.27	-1.536	0.136
	No	19	6.21	2.07		
Edge number	Yes	11	10.91	5.43	-0.562	0.578
	No	19	12.42	9.29		
Density	Yes	11	0.93	0.12	2.342	0.027
	No	19	0.76	0.27		
Betweenness centralization	Yes	11	0.03	0.05	-2.140	0.045
	No	19	0.14	0.23		
(c) Public-private partnership projects						
	Within budget	N	Mean	Std. Deviation	t	Sig.
Network size	Yes	6	7.00	3.16	-0.632	0.558
	No	3	8.33	2.89		
Edge number	Yes	6	22.67	26.38	1.460	0.200
	No	3	6.67	3.51		
Density	Yes	6	0.75	0.30	4.113	0.006
	No	3	0.23	0.07		
Betweenness centralization	Yes	6	0.28	0.32	-0.322	0.757
	No	3	0.33	0.15		

### 3.5 Node-level comparison results

We then conducted node-level centrality comparisons across the three aggregated stakeholder networks. Table 5 shows the top-5 stakeholders according to this study’s three centrality measures. According to the degree centrality measure, from Table 5(A), there are three common stakeholders (project team, clients and construction manager) between public and private projects, one (project sponsor) between public and PPP, and two (project manager and architects) between private and PPP.

**Table 5:** Top-5 nodes or stakeholders in the three different aggregated networks.

(a) Based on the degree centrality						
**Rank**	**Public**		**Private**		**Public-private partnership**	
	*Stakeholder name*	*Value*	*Stakeholder name*	*Value*	*Stakeholder name*	*Value*
1	Project Team	0.126	Project Manager	0.238	Civil Engineer	0.278
2	Clients	0.084	Project Team	0.178	Architects	0.270
3	Construction Manager	0.057	Clients	0.158	Project Manager	0.266
4	Construction Contractor	0.054	Architects	0.156	Project Sponsor	0.242
5	Project Sponsor	0.050	Construction Manager	0.124	Environmental Regulators	0.234
(b): Based on the closeness centrality						
**Rank**	**Public**		**Private**		**Public-private partnership**	
	*Stakeholder name*	*Value*	*Stakeholder name*	*Value*	*Stakeholder name*	*Value*
1	Clients	0.813	Project Manager	0.850	Building Control/Building Regulators	0.700
2	Project Team	0.780	Project Team	0.791	Environmental Regulators	0.667
3	Construction Manager	0.722	Architects	0.739	Architects	0.667
4	Architects	0.709	Clients	0.694	Project Manager	0.651
5	Project Sponsor	0.709	Construction Manager	0.694	Managing Director	0.651
(c): Based on the betweenness centrality						
**Rank**	**Public**		**Private**		**Public-private partnership**	
	*Stakeholder name*	*Value*	*Stakeholder name*	*Value*	*Stakeholder name*	*Value*
1	Clients	0.201	Project Manager	0.181	Project Manager	0.220
2	Project Team	0.130	Project Team	0.141	Government	0.146
3	Project Sponsor	0.106	Architects	0.091	Building Control/Building Regulators	0.143
4	Civil Engineer	0.059	Clients	0.057	Architects	0.142
5	Construction Manager	0.057	Construction Manager	0.042	Regulatory Authorities	0.140

In terms of the closeness centrality measure, the stakeholder role of ’architects’ is in the top-5 lists of all three project types, as displayed in Table 5(B). Although they appeared in a different order, public and private projects have almost identical top-5 stakeholder lists for this measure, except for one stakeholder role. The public and PPP lists have only one common stakeholder (architects), and there are two common stakeholders (project manager and architects) between private and PPP top-5 lists.

There is no common stakeholder among the top-5 stakeholders across three project types for the betweenness centrality measure like the degree centrality measure. Three stakeholders (clients, project team and construction manager) are common between public and private lists. Project managers and architects are the common ones between private and PPP lists. And there is no common stakeholder between public and PPP lists.

In summary, a few stakeholders commonly appeared in different lists of Table 5(A)–5(C). However, there is a striking difference among the top-5 lists of stakeholders based on the three centrality measures.

**Fig 7:**
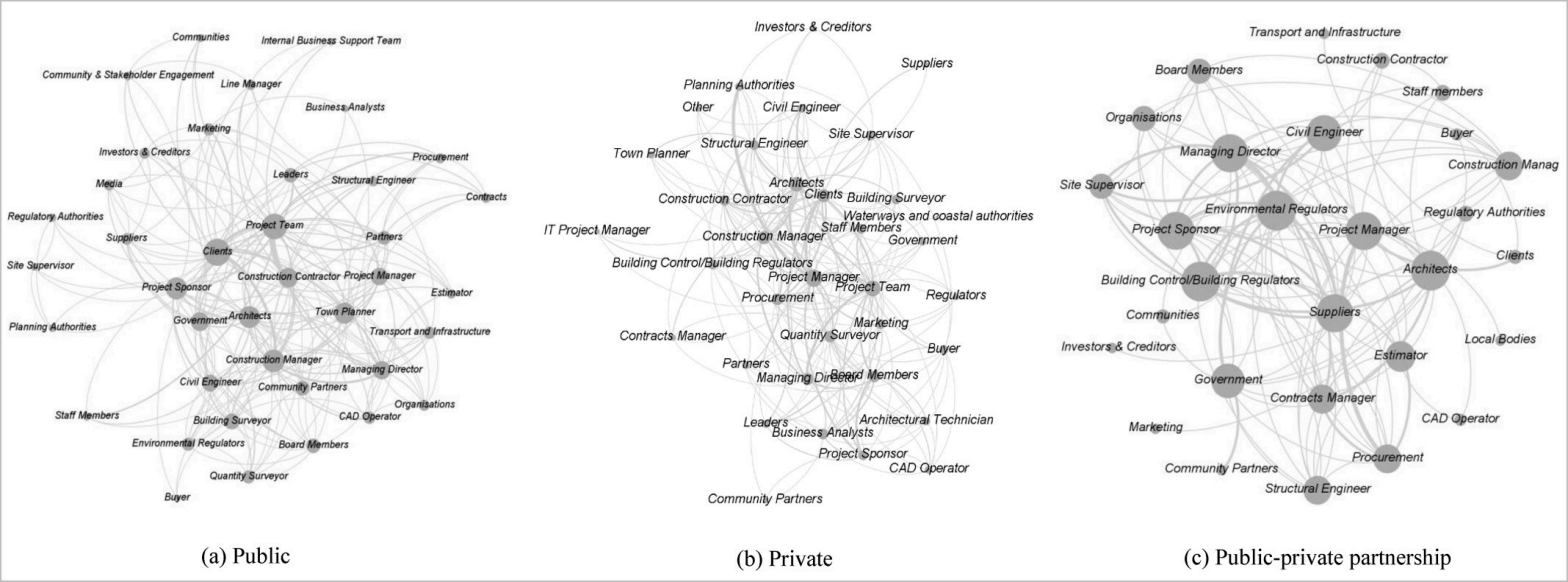
Visualizations of the three aggregated networks: (a) public, (b) private, and (c) public-private partnership. The size of a node proportionates to its degree centrality value. The thickness of an edge proportionates to its edge weight.

The visualizations in Fig 7 of these three aggregated networks further reflect the results reported in Table 5.

### 3.6 Exponential random graph model outcome

The ERGM estimations of the three aggregated stakeholder networks show a substantial difference in their convergence and effect statistics. The aggregated public and private networks had converged. We attempted several combinations of different effects, but the aggregated PPP network converged only for the edge effect.

As in Table 6(A), we considered six effects for the aggregated public network. They are edge, star2, star3, triangle, isolates and isolate-edges. The absolute value of the t-ratio for each of these effects is less than 0.1, indicating good model convergence [47]. The absolute value of the ratio between parameter and standard error is less than two for each of these effects, indicating their insignificant presence or absence in the aggregated public network [48].

**Table 6:** The estimation results from the exponential random graph model: (a) aggregated public network; and (b) the aggregated private network. The aggregated PPP network does not converge. AT stands for ’*alternative-k-triangles*’.

(a) Aggregated public network				
**Effects**	**Parameter**	**Standard error**	**t-ratio**	$\left|\frac{Parameter}{Standard\;error}\right|$
Edge	-2.2882	1.951	-0.004	1.173
Star2	-0.0777	0.565	0.007	0.138
Star3	0.0075	0.19	0.014	0.039
Triangle	0.6594	0.575	0.041	1.147
Isolates	1.3238	1.339	0.028	0.989
Isolate-edges	0.9309	1.324	0.098	0.703
(b) Aggregated private network				
**Effects**	**Parameter**	**Standard error**	**t-ratio**	$\left|\frac{Parameter}{Standard\;error}\right|$
Edge*	-2.4662	1.115	-0.086	2.212
Star2	-0.0318	0.544	-0.083	0.058
Star3	-0.0047	0.362	-0.062	0.013
Star5	-0.0368	0.285	0	0.129
Isolate-edges	0.8889	1.149	0.078	0.774
AT	0.1949	0.253	-0.035	0.770
(c) Aggregated public-private partnership network				
**Effects**	**Parameter**	**Standard error**	**t-ratio**	$\left|\frac{Parameter}{Standard\;error}\right|$
Edge*	-5.3621	0.756	0.095	7.093

On the other hand, as illustrated in Table 6(B), the aggregated private stakeholder network has been converged with edge, star2, star3, star5, isolate-edges and AT (alternative-k-triangles) effects. Four (Edge, Star2, Star3 and IsolateEdges) of these six effects are common with the public network estimation results from Table 6(A). Like the aggregated public network, all t-ratio values are less than 0.10, indicating a good model fit. Notably, the edge effect has a negative parameter value, and the absolute value of the ratio of its parameter and standard error values is higher than two. This means that when controlling for other included effects in the model, a pair of stakeholder roles in the aggregated private network is more likely not to be connected than the presence of a connection between them, which was not the case in the public network.

The result for the aggregated PPP network is presented in Table 6(C). This network converged only for the edge effect, which shows a significant impact with a t-ratio value of less than 0.10.

We further investigate the effects of different motifs. For this, we first fix the underlying effect to 0. If this setting significantly worsens the goodness-of-fit (GOF) of the model’s simulated motifs as compared to their observed distributions in the surveyed networks (i.e., their t-ratio values become well above 0.10), then the effect can be considered an important effect for the network. We tested different combinations of the underlying motifs. We found that the triangle effect plays an important role in the aggregated public network through this process. AT and star2 play a similarly important role in the aggregated private network.

In summary, both aggregated public and private networks converged with six effects. Two effects (triangle and isolates) impact only the convergence of the aggregated public network. Likewise, two other effects (star5 and AT) affect only the convergence of the aggregated private network. Most importantly, the edge effect played a significantly negative role in the aggregated private network; however, it did not play a similar role for the aggregated public network. The GOF analyses reveal that the triangle effect plays an important role in the convergence of the aggregated public network. AT and star2 play a similar role in the aggregated private network on the other side.

## 4. Discussion

Among the 56 survey responses, 17 are from public projects, 30 from private projects, and nine from PPP projects. This response statistic aligns with the fact that most projects are generally private, followed by the public and PPP categories, except for a few exceptions in a few industries (e.g., construction domain). In 1999, private projects totalled more than three-quarters of US construction spending, and the remaining one-quarter was spent on public and PPP projects [49]. Although the PPP model has gained considerable attention since then for implementing different projects, the overall spending in this sector is still relatively low compared with the public and private sectors [10].

Among the four top-5 lists for each of the three project categories from Tables 2 and 5(A)–5(C), there is a robust intra-group homogeneity and inter-group heterogeneity. As illustrated in Table 7, four stakeholders are common for the public project category. They are clients, construction manager, project sponsor and project team. All four lists for the private project category have the same five stakeholders but different orders. On the other side, the PPP category has two stakeholders in common (architects and project manager) across its four top-5 lists. This shows a solid intra-group homogeneity for all three project categories. However, no stakeholder is common across these three groups, a vital sign of inter-group heterogeneity. Between the public and private groups, there are three stakeholders in common (clients, construction manager and project team), a piece of evidence of inter-group homogeneity between them. Similarly, architects and project manager are the common stakeholders between private and PPP categories. Public and PPP categories do not have any such common stakeholder.

**Table 7:** Common stakeholders among the four top-5 lists (for the public, private and public-private partnership project categories) from Tables 2 and 5(A)–5(C). We follow the alphabetical order in placing them on the table.

Public	Private	Public-private partnership
Clients	Architects	Architects
Construction Manager	Clients	Project Manager
Project Team	Construction Manager	---
Project Sponsor	Project Manager	---
---	Project Team	----

Overall, the PPP networks are the most centralized among the three network types according to each of the three applied centralization measures (see average centralization values for the PPP network in Table 1). According to the definition provided in section 2.2 (Network measures), each of the three centralization measures (degree centralization, closeness centralization and betweenness centralization) has a maximum value of 1 for a star graph (Fig 2(B)). Thus, on average, PPP networks tend to form a more star-like network structure than the other two network types (public and private). In a star graph, one node is in the central position, and the other remaining network nodes are connected only to this central node. The remaining nodes depend on the central node to make any communications among themselves. It means that, in PPP networks, there is the highest disparity between the most centrally prominent actors and the rest of the stakeholders that revolve around them in the project.

Since all centralization measures consistently pointed to PPP projects as being most centralized, it is important to explore which stakeholders drove the PPP network centralization. The numerator of each of the three centralization measures (Eqs 2, 4 and 6) considers the difference between the highest observed corresponding centrality value and the centrality value for each of the remaining network nodes. Thus, the centrality values of Table 5(A)–5(C) can provide information about which stakeholders drove the PPP network centralization. Apart from the frequently appearing PPP stakeholders that also often engage in the other two project types (i.e. architects, project sponsor and project manager), few other stakeholders were solely found in the top-5 centrality lists for PPP projects. They are civil engineer, environmental regulators, building control/building regulators, managing director, government and regulatory authorities. Although minimum centrality values of other stakeholders could contribute to the centralization value, the centrality values of these stakeholders positively impact the centralization scores of PPP networks.

At the project level, the sheer number of links (captured by the density measure) in a stakeholder network is clearly associated with the financial performance in projects of all types (Table 4). This association is statistically significant at p<0.05 for private and PPP projects. A higher number of relative links enables more engagement between stakeholders. As evidenced in the literature, a higher stakeholder engagement improved project performance through achieving sustainability [1, 50].

The ERGM analysis further suggested that after accounting for potential endogenous effects, there is a generally negative propensity to form partnerships across the significantly larger pools of diverse stakeholders in public projects. By contrast, such a potentially negative networking tendency was not systematically observed in the smaller stakeholder networks of the private sector projects. It is worth noting that these differences were not noticed in the descriptive data as the simple network density statistics in the two sectors are identical, and the basic average number of links among public sector project stakeholders is more than double that of private-sector project stakeholders (Table 1).

ERGM can consider endogenous network processes commonly observed in real-world networks, such as partners of partners being more likely to become network partners with each other or central network actors being more attractive targets of other actors’ networking activities. Effects that represent these tendencies proved necessary to include for the ERGM model convergence for the public and private projects. Although they were not significant in terms of the usual “parameter divided by standard error” heuristic, one possible interpretation of the negative edge findings is that partnerships among stakeholders in large public projects are less likely if the stakeholders do not have any partner in common in the project network. The interpretation is consistent with the results of the sensitivity test, where fixing the triangle parameter to zero in the public network significantly worsened the model fit. In other words, while controlling for other potential network tendencies, it does seem that stakeholders in public project networks do tend to create network relationships preferentially by linking with partners of existing partners (and thus creating triangle network structures) rather than project stakeholders with no partners in common.

The PPP model also shows a significantly negative edge effect, which would be expected given the network density and presence of no other effects in this basic model. The density of the PPP networks is also lower than that of other project types. Endogenous effects were not possible to include in the PPP projects, resulting in an inadequate model fit, particularly in the triangle and different star structures. This suggests that such structures are significantly represented in the real networks and not adequately captured in the too basic but only converged model for PPP.

This study has some limitations. Like in most retrospective survey studies, the data may be a subject to participants’ recall bias, respondents may forget some actors or relationships despite the questionnaire prompts [51]. As much as practicable, usual measures have been taken to mitigate recall errors and other respondents’ biases (for example, by withholding the research hypotheses from them not to influence their answers) and to increase the comparability between the respective respondents’ projects by focusing only on construction projects. This preference of comparability comes with a natural trade off with external generalizability and caution should be applied when extending the present results beyond this sample, especially to projects in other industries. Further investigations should explore stakeholder networks in other types of projects and ideally include a longitudinal component to better explain the dynamic interplays between stakeholder network evolution and project progression.

## 5. Conclusion

This study found significant differences among public, private and PPP projects in terms of their stakeholder networks. First, the lists of top-5 stakeholders that appeared most frequently in the project networks is different. Second, there is a statistically significant difference in network-level measures of network size, edge number, density and betweenness centralization across these three project categories. Among these four measures, density further reveals a significant difference between within-budget and cost overrun projects for private and PPP project categories. Third, there is a striking difference among the top-ranked stakeholders based on the three network centrality measures in the aggregated networks. Finally, ERGM estimations reveal differences in convergence and effect statistics among them.

Although social network analysis is emerging as an important approach in studying stakeholder relationships [52], previous studies of stakeholder networks have often emphasized actor interactions at dyadic and node levels [e.g., 2, 25] or focused on fragmented or isolated case studies [53] or were inferred from secondary data from reports that were not compiled for network analysis or were based on other proxies [34]. Moreover, while strong conceptual and theoretical developments have been made in adjacent fields [54], theoretical developments in the study of stakeholder networks surrounding projects have been limited to fewer authors. Theoretical developments usually tend to progress hand-in-hand with empirical advances and the availability of more comprehensive data. Despite the clear merits of previous works cited in the introduction and just above, the number of empirical studies comparing entire stakeholder network structures across statistically analysable higher number of cases has remained limited and the data that would allow researchers such explorations have been scarce [55, 56]. Therefore, our understanding of how stakeholder networks form around different types of projects has been limited. Working with a larger number of consistently collected stakeholder network data that includes the stakeholders’ perceptions of their networks, this study applied dyadic, node-level and, importantly, network-level measures (e.g., centralization measures) and sophisticated network models (exponential random graph models) for comparing the network structures found within the different sectors of project networks. Such an application of network measures and models enables researcher a deeper comparison of the structures of different types of stakeholder networks, which is a contribution of this study to this stream of literature.

In addition to revealing significant differences in the patterns of stakeholder engagement across public, private and public-private partnership projects, this study demonstrates some of the myriads of possibilities and opportunities for further research on stakeholder engagement using methods of network research. Firstly, this study is a cross-sectional analysis of projects, and longitudinal investigations would allow one to better understand stakeholder networks within different project phases and their impacts on performance. Secondly, the results have highlighted the variation between stakeholder network structures in different project types. Further exploration is needed to better understand the drivers and consequences of these trends. Finally, stakeholder engagement and management policies continually change within various industry contexts. Therefore, the findings must be interpreted in the contemporary context, whether the shifting contractual landscape of public projects and the economically changing context within private projects.

## Supporting information

S1 Table (a): Stakeholder list according to the survey design & (b): Summarized stakeholder list.
(PDF)

S1 File The design of the survey questionnaire. (DOCX)

## References

[1] Bal M., Bryde D., Fearon D., and Ochieng E., Stakeholder engagement: Achieving sustainability in the construction sector. Sustainability, 2013. 5(2): p. 695–710. doi: 10.3390/su5020695

[2] Rowley T.J., Moving beyond dyadic ties: A network theory of stakeholder influences. Academy of management Review, 1997. 22(4): p. 887–910.

[3] Greenwood M., Stakeholder engagement: Beyond the myth of corporate responsibility. Journal of Business ethics, 2007. 74(4): p. 315–327.

[4] Karlsen J.T., Project stakeholder management. Engineering Management Journal, 2002. 14(4): p. 19–24.

[5] Eskerod P., Huemann M., and Savage G., Project stakeholder management—Past and present. Project management journal, 2015. 46(6): p. 6–14.

[6] Zaharna R.S., The public diplomacy challenges of strategic stakeholder engagement, in Trials of Engagement. 2011, Brill Nijhoff. p. 201–229. doi: 10.1163/ej.9789004179400.i-309.52

[7] Mathur V.N., Price A.D., and Austin S., Conceptualizing stakeholder engagement in the context of sustainability and its assessment. Construction Management and Economics, 2008. 26(6): p. 601–609. doi: 10.1080/01446190802061233

[8] Tabish S. and Jha K., Identification and evaluation of success factors for public construction projects. Construction management and economics, 2011. 29(8): p. 809–823.

[9] Assbeihat J.M., Factors affecting delays on private construction projects. Technology, 2016. 7(2): p. 22–33.

[10] Wang H., Xiong W., Wu G., and Zhu D., Public–private partnership in Public Administration discipline: a literature review. Public management review, 2018. 20(2): p. 293–316.

[11] Tang L., Shen Q., and Cheng E.W., A review of studies on public–private partnership projects in the construction industry. International journal of project management, 2010. 28(7): p. 683–694.

[12] Oppong G.D., Chan A.P., and Dansoh A., A review of stakeholder management performance attributes in construction projects. International journal of project management, 2017. 35(6): p. 1037–1051.

[13] Freeman R.E., Strategic management: A stakeholder approach. 2010: Cambridge university press.

[14] Donaldson T. and Preston L.E., The stakeholder theory of the corporation: Concepts, evidence, and implications. Academy of management Review, 1995. 20(1): p. 65–91.

[15] Jones T.M. and Wicks N.C., Convergent stakeholder theory, in Business Ethics and Strategy. 2018, Routledge. p. 361–376.

[16] Freeman R.E., Divergent stakeholder theory. Academy of management review, 1999. 24(2): p. 233–236.

[17] Friedman A.L. and Miles S., Developing stakeholder theory. Journal of management studies, 2002. 39(1): p. 1–21.

[18] Laplume A.O., Sonpar K., and Litz R.A., Stakeholder theory: Reviewing a theory that moves us. Journal of management, 2008. 34(6): p. 1152–1189.

[19] Roulet T.J. and Bothello J., Tackling grand challenges beyond dyads and networks: Developing a stakeholder systems view using the metaphor of ballet. Business Ethics Quarterly, 2021: p. 1–31.

[20] Fares J., Chung K.S.K., and Abbasi A., Stakeholder theory and management: Understanding longitudinal collaboration networks. Plos one, 2021. 16(10): p. e0255658. 34648505 10.1371/journal.pone.0255658 PMC8516199

[21] Parmar B.L., Freeman R.E., Harrison J.S., Wicks A.C., Purnell L., and De Colle S., Stakeholder theory: The state of the art. Academy of Management Annals, 2010. 4(1): p. 403–445.

[22] Freeman R.E., Phillips R., and Sisodia R., Tensions in stakeholder theory. Business & Society, 2020. 59(2): p. 213–231.

[23] Turner J.R. and Müller R., On the nature of the project as a temporary organization. International journal of project management, 2003. 21(1): p. 1–8.

[24] Littau P., Jujagiri N.J., and Adlbrecht G., 25 years of stakeholder theory in project management literature (1984–2009). Project Management Journal, 2010. 41(4): p. 17–29.

[25] Chung K.S.K. and Crawford L., The role of social networks theory and methodology for project stakeholder management. Procedia-Social and Behavioral Sciences, 2016. 226: p. 372–380.

[26] Yang J., Shen Q., and Ho M., An overview of previous studies in stakeholder management and its implications for the construction industry. Journal of facilities management, 2009.

[27] Mohan V. and Paila A.R., Stakeholder Management in Infrastructure/Construction Projects: The Role Of Stakeholder Mapping And Social Network Analysis (SNA). Aweshkar Research Journal, 2013. 15(1).

[28] Beach S., Keast R., and Pickernell D., Unpacking the connections between network and stakeholder management and their application to road infrastructure networks in Queensland. Public Management Review, 2012. 14(5): p. 609–629.

[29] Freeman R.E., Strategic management: A stakeholder approach. 1984: Cambridge university press.

[30] Missonier S. and Loufrani-Fedida S., Stakeholder analysis and engagement in projects: From stakeholder relational perspective to stakeholder relational ontology. International journal of project management, 2014. 32(7): p. 1108–1122.

[31] Okazaki S., Plangger K., Roulet T., and Menéndez H.D., Assessing stakeholder network engagement. European Journal of Marketing, 2020.

[32] Vance‐Borland K. and Holley J., Conservation stakeholder network mapping, analysis, and weaving. Conservation Letters, 2011. 4(4): p. 278–288.

[33] Yang R.J. and Zou P.X., Stakeholder-associated risks and their interactions in complex green building projects: A social network model. Building and environment, 2014. 73: p. 208–222.

[34] Guo X. and Kapucu N., Examining stakeholder participation in social stability risk assessment for mega projects using network analysis. International Journal of Disaster Risk Management, 2019. 1(1): p. 1–31.

[35] Pinheiro M.L., Serôdio P., Pinho J.C., and Lucas C., The role of social capital towards resource sharing in collaborative R&D projects: Evidences from the 7th Framework Programme. International Journal of Project Management, 2016. 34(8): p. 1519–1536.

[36] Snijders T.A.B., Pattison P.E., Robins G.L., and Handcock M.S., New specifications for exponential random graph models. Sociological Methodology, 2006. 36(1): p. 99–153.

[37] Stohl C., Giles H., and Maass A., Social networks and intergroup communication, in In H. Giles & A. Maass (Eds.) Advances in intergroup communication. 2016, Peter Lang Publishing. p. 317–339.

[38] Krosnick J.A., Questionnaire design, in The Palgrave handbook of survey research. 2018, Springer. p. 439–455.

[39] Contandriopoulos D., Larouche C., Breton M., and Brousselle A., A sociogram is worth a thousand words: proposing a method for the visual analysis of narrative data. Qualitative Research, 2018. 18(1): p. 70–87.

[40] Wasserman S. and Faust K., Social network analysis: Methods and applications. 2003, Cambridge: Cambridge University Press.

[41] Denver Foundation. Identifying Internal and External Stakeholders. 2022 [cited 2022 21 March]; Available from: http://www.nonprofitinclusiveness.org/identifying-internal-and-external-stakeholders.

[42] Stakeholdermap.com. Stakeholder Analysis, Project Management, templates and advice. 2022; Available from: https://www.stakeholdermap.com/stakeholders-construction.html.

[43] Field A., Discovering statistics using SPSS. 2009: Sage Publications Ltd.

[44] Hunter D.R., Handcock M.S., Butts C.T., Goodreau S.M., and Morris M., ergm: A package to fit, simulate and diagnose exponential-family models for networks. Journal of statistical software, 2008. 24(3): p. nihpa54860. 19756229 10.18637/jss.v024.i03 PMC2743438

[45] Wang P., Robins G., and Pattison P., PNet: a program for the simulation and estimation of exponential random graph models. Melbourne School of Psychological Sciences, The University of Melbourne, 2009.

[46] Terrell G.R. and Scott D.W., Variable kernel density estimation. The Annals of Statistics, 1992. 20(3): p. 1236–1265.

[47] Wang P., Robins G., Pattison P., and Lazega E., Exponential random graph models for multilevel networks. Social Networks, 2013. 35(1): p. 96–115.

[48] Matous P., Wang P., and Lau L., Who benefits from network intervention programs? TERGM analysis across ten Philippine low-income communities. Social Networks, 2021. 65: p. 110–123.

[49] Hwang B.-G., Liao P.-C., and Leonard M.P., Performance and practice use comparisons: Public vs. Private owner projects. KSCE Journal of Civil Engineering, 2011. 15(6): p. 957–963.

[50] Menoka B., Stakeholder engagement and sustainability-related project performance in construction. 2014: Liverpool John Moores University (United Kingdom).

[51] Bethlehem J., Selection bias in web surveys. International statistical review, 2010. 78(2): p. 161–188.

[52] Nguyen T.S., Mohamed S., and Panuwatwanich K., Stakeholder Management in Complex Project: Review of Contemporary Literature. Journal of Engineering, Project & Production Management, 2018. 8(2).

[53] Uddin S., Social network analysis in Project management–A case study of analysing stakeholder networks. The Journal of Modern Project Management, 2017. 5(1).

[54] Prell C., Hubacek K., and Reed M., Stakeholder analysis and social network analysis in natural resource management, in Handbook of applied system science. 2016, Routledge. p. 367–383.

[55] Zheng X., Le Y., Chan A.P., Hu Y., and Li Y., Review of the application of social network analysis (SNA) in construction project management research. International journal of project management, 2016. 34(7): p. 1214–1225.

[56] Gan X., Chang R., and Wen T., Overcoming barriers to off-site construction through engaging stakeholders: A two-mode social network analysis. Journal of Cleaner Production, 2018. 201: p. 735–747.

