# Supplementary material for: Stakeholder engagement variability across public, private and public-private partnership projects: A data-driven network-based analysis
Source: PLoS One. 2023 Jan 6;18(1):e0279916. doi: 10.1371/journal.pone.0279916 (PMC9821786; doi:10.1371/journal.pone.0279916)
Supplement: S1 Table — (a): Stakeholder list according to the survey design & (b): Summarized stakeholder list. (PDF) [file pone.0279916.s001.pdf]

Supplementary Table 1(a): Stakeholder list according to the survey design

Stakeholder List

**External**	**Private**	**Professional**	Construction Contractor
			Architects
			Quantity Surveyor
			Building Surveyor
			Construction Manager
			Suppliers
			Marketing
			Procurement
			Civil Engineer
			Architectural Technician
			CAD operator
			Estimator
			Structural Engineer
		**Non-Professional**	Donors
			Buyer
			Clients
	**Public**	**Professional**	Town Planner
			Regulatory authorities
			Environmental regulators
			Planning authorities
			Building Control/Building Regulations
			Transport and Infrastructure
			Waterways and coastal authorities
			Government
			Regulators
			Trade associations
		**Non-Professional**	Volunteers
			Donors
			Community partners
			Pressure groups
			Communities
			Organisations
			Media
			Local Communities
			Clients

**Internal**	**Private**	**Professional**	Board members
			Staff members
			Leaders
			Project Manager
			Site Supervisor
			Managing Director
			Contracts Manager
			Partners
			Project Team
			Business Analysts
			Architects
			Structural Engineer
			Civil Engineer
		**Non-Professional**	Investors & Creditors
			Project Sponsor
	**Public**	**Professional**	Volunteers
		**Non-Professional**	Volunteers
			Project Sponsor

Supplementary Table 1(b): Summarized stakeholder list

Stakeholder Category (Summary)
Board members
Staff members
Volunteers
Donors
Clients
Community partners
Leaders
Construction Contractor
Project Manager
Architects
Site Supervisor
Structural Engineer
Quantity Surveyor
Building Surveyor
Construction Manager
Town Planner
Managing Director
Contracts Manager
Suppliers
Pressure groups
Trade associations
Regulatory authorities
Marketing
Procurement
Civil Engineer
Architectural Technician
Buyer
CAD operator
Estimator
Environmental regulators
Planning authorities
Building Control/Building Regulations
Transport and Infrastructure
Waterways and coastal authorities
Government
Investors & Creditors
Communities
Organisations
Partners
Media
Regulators
Local Communities
Project Team
Business Analysts
Project Sponsor
