# Supplementary material for: Stakeholder engagement variability across public, private and public-private partnership projects: A data-driven network-based analysis
Source: PLoS One. 2023 Jan 6;18(1):e0279916. doi: 10.1371/journal.pone.0279916 (PMC9821786; doi:10.1371/journal.pone.0279916)
Supplement: S1 File — (DOCX) [file pone.0279916.s002.docx]

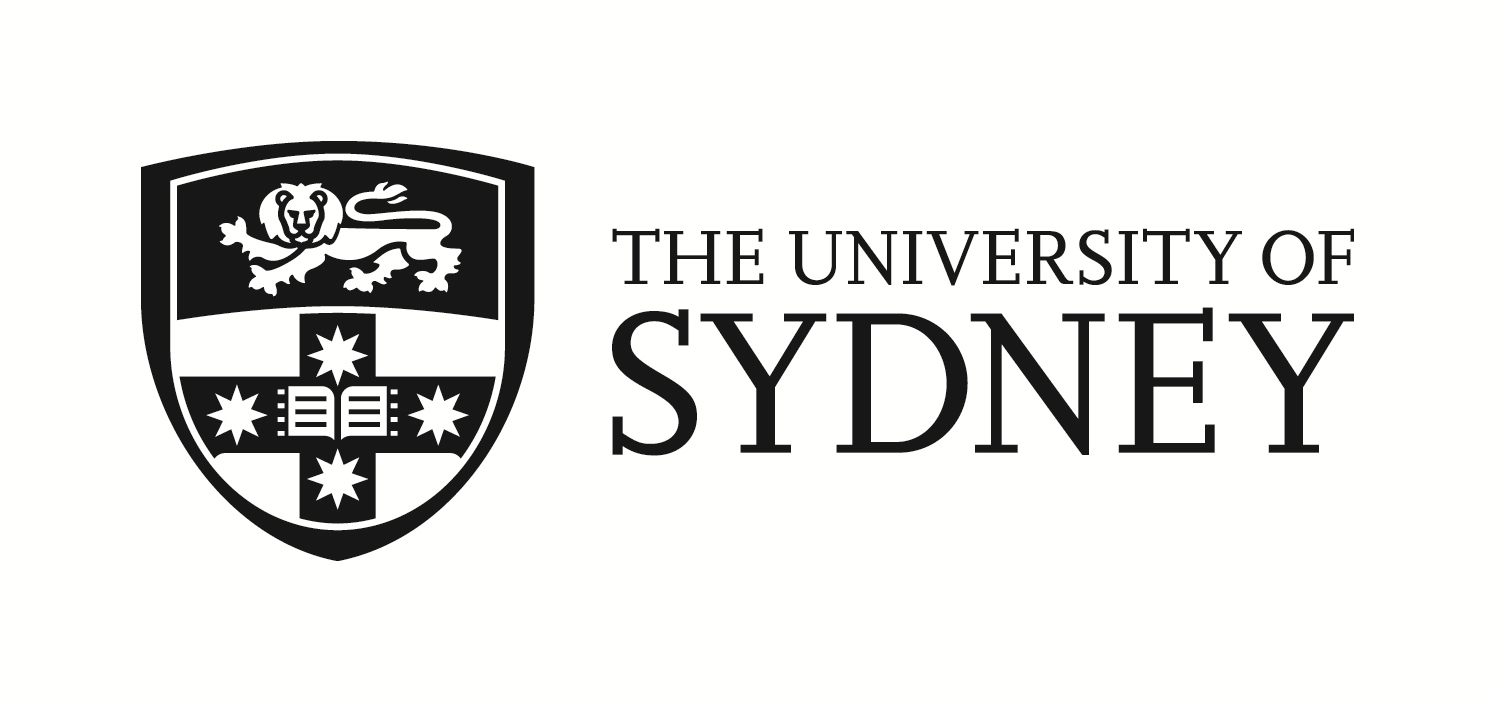


**Faculty of Engineering and Information Technologies**

| Dr Shahadat Uddin  E-mail [shahadat.uddin@sydney.edu.au](mailto:shahadat.uddin@sydney.edu.au)  Telephone: +61 2 9351 2118 | Stephen Ong  E-mail song1382@uni.sydney.edu.au  Mobile (0451) 213 348 | SIT Building J12  Address 1 Cleveland Street,  Darlington, NSW, 2008 |
| --- | --- | --- |

**Participant Information Sheet-**

**Determining the Components, Contexts and Network Structures For Major Projects**

Project managers are an important component and play a pivotal role in the functioning, execution and success of projects as well as megaprojects. Project managers engage in an exchange of numerous relationships with various members and stakeholders throughout a project. Despite this, an understanding of these relationships remains largely limited. Further investigation will yield a more in-depth level of understanding and allow project managers to develop greater control.

This questionnaire aims to assist project managers in developing this understanding of stakeholder networks and the environments which promote and allows the incubation of successful projects. This is to be accomplished through the cross-comparison of projects.

This questionnaire has been designed to be succinct and will take approximately 15-30 minutes of your time. Further to this, all responses will remain anonymous and confidential throughout the entire research process and because of this, your honest answers are most appreciated.

This project has been approved by the University of Sydney’s Human Ethics Research Committee (2019/794). The responses you provide have the potential to contribute to and provide positive impact on the quality of management within projects, and in particular megaprojects. The completion and return of this survey communicates your consent to participate in this research study and survey. Despite not being obligated to participate, declining to participate will in not affect you whatsoever.

We thank you in advance for your contribution and will provide a report outlining findings when cross-compared with other projects. It is aimed that these findings will be able to contribute to the planning, execution as well as management of future projects.

We greatly appreciate your help.

Kind Regards*,*

Stephen

**The following preliminary questions pertain to the most recent project of significant monetary value (defined in this context as minimum AUD$1 million)**

| Project Name | ……………………………………………………………………….. |
| --- | --- |
| Project Description (in brief) | ……………………………………………………………………….. |
| What was the monetary value of this component? | ……………………………………………………………………….. |
| Analytical Hierarchy Process (AHP) (Saaty, 2008)  Analytical Hierarchy Process is a decision-making process utilised by project managers in a decision-making process. In order to understand the parameters of the project, please construct a general AHP mind-map incorporating the key elements underlying the main aim of the project. |  |
| 1. What was the main purpose of the project? | ……………………………………………………………………….. |
| 1. What component of the project were you managing/ responsible for? | ……………………………………………………………………….. |
| 1. What was the project type (Private/ Public/ Joint)? | ……………………………………………………………………….. |
| 1. In which countries was the project hosted/ executed? | ……………………………………………………………………….. |
| 1. Were you required to correspond with any professional(s) overseas? | ………………………………………………………………………. |
| 1. Please fill in the following regarding the contract: | Contract Date:  ………………………………………………………………………..  Contract Type:  ………………………………………………………………………..  Contract Owners/ joint venture Partners:  ………………………………………………………………………..  Contract Price:  ……………………………………………………………………….. |
| 1. What types of technical expertise were required (please list)? | ……………………………………………………………………….. |
| 1. What were some relevant technical skills and experience you found helpful in managing the project (please list)? | ……………………………………………………………………….. |
| 1. What was one factor you found challenging about the project? | ……………………………………………………………………….. |
| 1. Where was the funding for the project sourced from? | ……………………………………………………………………….. |
| 1. Who are the primary beneficiaries of this project? | ……………………………………………………………………….. |
| 1. When was the project completed? | (YYYY) |
| 1. What was the total duration of the project? | …………………………………………………………….…..Month(s)  …………………………………………………………………Year(s) |
| 1. Was this project completed on time, budget and of an expected quality? (Please circle Yes/ No) | \| **Time** \| Yes /No \| \| --- \| --- \| \| **Budget** \| Yes/ No \| \| **Quality** \| Yes/ No \| |

**Part A. Background Socio-Demographic Survey**

*This section asks some general demographic question about yourself. Please fill in the responses in the right-hand column.*

| Demographic Questions | Answers |
| --- | --- |
| 1. Please state your industry | ……………………………………………………………………….. |
| 1. Please state your occupation | ……………………………………………………………………….. |
| 1. How many years have you worked within this occupation? | Year(s) |
| 1. Please state the company you are currently working for | ……………………………………………………………………….. |
| 1. How many years have you worked within this role? | Year(s) |

**Part B. Professional Network Questions**

| “Professional Network” refers to the relevant professionals with whom you work and communicate with to achieve the desired results of the relevant project (for example designers, other relevant managers and so on). |
| --- |

*The following questions pertain to the most recent project of significant monetary value which you have worked on. Please identify up to 15 of the most important* *stakeholders within your network (both internal/ external to your team and/or company). Please also identify the most common communication mode (e.g. Skype, telephone and so on), their closeness and proximity.*

|  | Stakeholder Type  (reference to drop-down list) | Communication Mode | Closeness | Proximity |
| --- | --- | --- | --- | --- |
| Person 1 |  |  |  |  |
| Person 2 |  |  |  |  |
| Person 3 |  |  |  |  |
| Person 4 |  |  |  |  |
| Person 5 |  |  |  |  |
| Person 6 |  |  |  |  |
| Person 7 |  |  |  |  |
| Person 8 |  |  |  |  |
| Person 9 |  |  |  |  |
| Person 10 |  |  |  |  |
| Person 11 |  |  |  |  |
| Person 12 |  |  |  |  |
| Person 13 |  |  |  |  |
| Person 14 |  |  |  |  |
| Person 15 |  |  |  |  |

| Code | Closeness |
| --- | --- |
| 1-5 | Frequent- Rare (Communication) |

| Code | Proximity |
| --- | --- |
| 1 | Works Closely (e.g., as a trainee) |
| 2 | Internal Team |
| 3 | Internal Company |
| 4 | External Company |
| 5 | External State |
| 6 | External Country |

***Section 1. Network Relationship Characteristics***

*This section is most pivotal and conducive to the analysis of your professional network. Please only complete for the unshaded areas of the matrix.*

*Answering method:*

1. *Please insert a ‘1’ firstly by row, for the stakeholders who have formed a relationship with another stakeholder/ person*
2. *Then by column, a ‘1’ also for the stakeholders who have formed a relationship with another stakeholder/ person*

|  | *Person 1* | *Person 2* | *Person 3* | *Person 4* | *Person 5* | *Person 6* | *Person 7* | *Person 8* | *Person 9* | *Person 10* | *Person 11* | *Person 12* | *Person 13* | *Person 14* | *Person 15* |
| --- | --- | --- | --- | --- | --- | --- | --- | --- | --- | --- | --- | --- | --- | --- | --- |
| *Person 1* |  |  |  |  |  |  |  |  |  |  |  |  |  |  |  |
| *Person 2* |  |  |  |  |  |  |  |  |  |  |  |  |  |  |  |
| *Person 3* |  |  |  |  |  |  |  |  |  |  |  |  |  |  |  |
| *Person 4* |  |  |  |  |  |  |  |  |  |  |  |  |  |  |  |
| *Person 5* |  |  |  |  |  |  |  |  |  |  |  |  |  |  |  |
| *Person 6* |  |  |  |  |  |  |  |  |  |  |  |  |  |  |  |
| *Person 7* |  |  |  |  |  |  |  |  |  |  |  |  |  |  |  |
| *Person 8* |  |  |  |  |  |  |  |  |  |  |  |  |  |  |  |
| *Person 9* |  |  |  |  |  |  |  |  |  |  |  |  |  |  |  |
| *Person 10* |  |  |  |  |  |  |  |  |  |  |  |  |  |  |  |
| *Person 11* |  |  |  |  |  |  |  |  |  |  |  |  |  |  |  |
| *Person 12* |  |  |  |  |  |  |  |  |  |  |  |  |  |  |  |
| *Person 13* |  |  |  |  |  |  |  |  |  |  |  |  |  |  |  |
| *Person 14* |  |  |  |  |  |  |  |  |  |  |  |  |  |  |  |
| *Person 15* |  |  |  |  |  |  |  |  |  |  |  |  |  |  |  |

**Part C. Project Information**

*In the following section, we would like to understand your project experience and background further.*

**The Crawford-Ishikura Factor Table for Evaluating Roles (CIFTER) (Aitken, Crawford, & Lille, 2007)**

*The Crawford-Ishikura Factor Table for Evaluating Roles (CIFTER) is a method employed by project managers in circumstances that have great complexity. Please circle in the following table to convey critical information about the project.*

| Project Management Complexity Factor | Descriptor and Points | | | |
| --- | --- | --- | --- | --- |
| 1. Stability of the overall project context | Very high (1) | High (2) | Moderate (3) | Low (4) |
| 2. Number of distinct disciplines, methods, or approaches involved in performing the project | Low (1) | Moderate (2) | High (3) | Very high (4) |
| 3. Magnitude of legal, social, or environmental implications from performing the project | Low (1) | Moderate (2) | High (3) | Very high (4) |
| 4. Overall expected financial impact (positive or negative) on the project’s stakeholders | Low (1) | Moderate (2) | High (3) | Very high (4) |
| 5. Strategic importance of the project to the organisation or organisations involved | Very low (1) | Low (2) | Moderate (3) | High (4) |
| 6. Stakeholder cohesion regarding the characteristics of the product of the project | Low (1) | Moderate (2) | High (3) | Very high (4) |
| 7. Number and variety of interfaces between the project and other organisational entities | Very low (1) | Low (2) | Moderate (3) | High (4) |

**Part D. Project Outcomes**

*A series of different parameters are usually measured to determine the performance of a project (Chan & Chan, 2004). Please fill in just the last two columns consisting of the project descriptors for us to determine the general project outcomes.*

*To assist with the response process, please note that only approximate responses are required for all of the following values.*

| Practical Completion Date: | Project Completion Date: | Construction Time  $\boldsymbol{Construction Time= Practical Completion Date}\boldsymbol{-}\boldsymbol{Project Commencement Date}$ |
| --- | --- | --- |
| Gross Floor Area (If applicable to building infrastructure projects): | **Construction Time:** | **Speed of Construction**  $Speed of Construction= \frac{Gross Floor Area (m^{2})}{Construction Time (In Days/ Weeks)}$ |
| Construction Time (if applicable to infrastructure projects): | **Revised Contract Period:** | **Time Variation**  $Time Variation= \frac{Construction Time - Revised Contract Period}{Revised Contract Period}X 100 Percent$ |
| Final Contract Sum: | **Gross Floor Area (if applicable to building infrastructure projects):** | **Unit Cost**  $Unit Cost= \frac{Final Contract Sum}{Gross Floor Area (m^{2})}$ |
| Net Value of Variations: | **Final Contract Sum:** | **Net Variation**  $NETVAR= \frac{Net Value of Variations}{Final Contract Sum}X 100 Percent$ |
| Total Number of Repeatable Construction Site Accidents (if applicable to infrastructure projects): | **Total Number of Workers Employed or Hours Worked (if applicable to infrastructure projects):** | **Health and Safety**  $Accident Rate= \frac{Total Number of Reportable Construction Site Accidents}{Total Number of Workers Employed or Hours Worked}X 1000$ |

**Please describe the following subjective measures of performance:**

| Environmental Performance |
| --- |
| Quality/ Functionality |
| Participant Satisfaction |
| User Satisfaction |

**Part E. In this section we would like to understand your attitude towards the management of large-scale projects.**

Please circle the most relevant and appropriate answer. Your honesty is most appreciated.

1. *Do you believe the management of team members constitutes the most challenging aspect of project management?*

**|===========|===========|===========|===========|==========|==========|**

| Strongly  Disagree | Agree | Slightly  Agree | Neither Agree Nor Disagree | Slightly  Disagree | Disagree | Strongly Disagree |
| --- | --- | --- | --- | --- | --- | --- |

1. *Do you believe that extremely large projects (for instance megaprojects), require an entirely different approach to management?*

**|===========|===========|===========|===========|==========|==========|**

| Strongly  Disagree | Agree | Slightly  Agree | Neither Agree nor Disagree | Slightly  Disagree | Disagree | Strongly Disagree |
| --- | --- | --- | --- | --- | --- | --- |

Project Views

*Please indicate in the following your agreement with the following statements by circling the statement that most accurately describes your view*

1. *Time zones form a barrier to effective communication*

**|===========|===========|===========|===========|==========|==========|**

| Strongly  Disagree | Agree | Slightly  Agree | Neither Agree Nor Disagree | Slightly  Disagree | Disagree | Strongly Disagree |
| --- | --- | --- | --- | --- | --- | --- |

1. *It is difficult to determine and quantify quality of the final product objectively*

**|===========|===========|===========|===========|==========|==========|**

| Strongly  Disagree | Agree | Slightly  Agree | Neither Agree Nor Disagree | Slightly  Disagree | Disagree | Strongly Disagree |
| --- | --- | --- | --- | --- | --- | --- |

| ***You have reached the end of this survey. Thank you so much!***  ***Please ensure that you have answered all questions.***  ***If you would like a report summary of the research findings, please fill in your contact details in the following:***  *First Name:*  *Surname:*  *Telephone:*  *Email:*  *Address:* |
| --- |

**Stephen Ong**

**School of Engineering and Information Technologies, J12**

**The University of Sydney**

**NSW 2006**

**References**

Aitken, A., Crawford, L., & Lille, E. (2007). *A study of project categorisation based on project management complexity.* Paper presented at the IRNOP VIII Conference (8th Annual International Research Network on Organizing by Projects).

Chan, A. P. C., & Chan, A. P. L. (2004). Key performance indicators for measuring construction success. *Benchmarking, 11*(2), 203-221.

Saaty, T. L. (2008). Decision making with the analytic hierarchy process. *International journal of services sciences, 1*(1), 83-98.
